# Discrimination of histopathologic types of childhood peripheral neuroblastic tumors based on clinical and biological factors

**DOI:** 10.1038/s41598-018-29382-x

**Published:** 2018-07-19

**Authors:** Shen Yang, Siyu Cai, Xiaoli Ma, Qi Zeng, Hong Qin, Wei Han, Xiaoxia Peng, Huanmin Wang

**Affiliations:** 1 Department of Surgical Oncology, Beijing Children’s Hospital, Capital Medical University, National Center for Children’s Health, Beijing, 100045 China; 2 Center for Clinical Epidemiology & Evidence-based Medicine, Beijing Children’s Hospital, Capital Medical University, National Center for Children’s Health, Beijing, 100045 China; 3 Hematology Oncology Center, Beijing Children’s Hospital, Capital Medical University, National Center for Children’s Health, Beijing, 100045 China; 4 Department of Thoracic Surgery, Beijing Children’s Hospital, Capital Medical University, National Center for Children’s Health, Beijing, 100045 China

## Abstract

The aim of this study was to discriminate the children malignant peripheral neuroblastic tumors (PNTs) from those with benign histotype ganglioneuroma (GN) based on clinical and biological characteristics in all PNTs. Four hundred and seventy-six patients were included in this study, containing 345 patients for model development and 131 patients for external validation. Multivariate logistic regression analysis was conducted to select potentially useful characteristics for discrimination of histopathology. External validation was performed for model evaluation. Compared with the main characteristics of GN (85/345, 24.6%), those of malignant PNTs (260/345, 75.4%) showed significant differences. Multivariate analysis was performed to further find the characteristics linked to histopathology. The results indicated that for the patients younger than 49 months, the primary site of adrenal and thoracic, the level of serum neuron-specific enolase (NSE) > 33 ng/mL, and tumor encasing blood vessels were the extremely important discrimination factors of malignant PNTs. The area under the receiver-operating-characteristic of the discrimination model was 0.96. The accuracy rate, sensitivity and specificity were 93.4%, 96.3% and 83.8%, respectively. Meanwhile, the accuracy rate of the external validation from the 131 patients was 97.0%. Overall, histopathologic type of childhood malignant PNTs can be discriminated based on age, primary site, NSE level and the relationship between primary tumor and blood vessels.

## Introduction

Peripheral neuroblastic tumors (PNTs) occur mostly in children and arise in cells derived from the neural crest. PNT is a heterogeneous disease, varying in terms of location, histopathologic appearance, biologic characteristics and prognosis. Histopathologic types of PNTs include the malignant histotypes neuroblastoma (NB), ganglioneuroblastoma nodular (GNBn), and the stroma-rich ganglioneuroblastoma intermixed (GNBi), and benign histotype ganglioneuroma (GN)^1^. Malignant PNTs, especially NB, account for most of PNTs, with nearly 50% of patients have a high risk phenotype characterized by widespread disease dissemination and poor long-term survival, are responsible for 12% of deaths associated with cancer in children younger than 15 years of age^2^.

Former studies have found that malignant PNTs differ from GN in that they affect younger children^3^, produce elevated amounts of catecholamines and other tumor markers^4^, and are often symptomatic^5,6^. For these patients with malignant PNTs, they need a comprehensive assessment of the disease and a multimodality therapy that usually includes chemotherapy, surgical resection, and even high-dose chemotherapy with hematopoietic stem-cell rescue, radiation therapy and immunologic therapy according to the risk group classification^7^. On the contrary, surgical resection or observation strategy due to the significant morbidity associated with surgery are the common therapies for GNs^8^.

For a patient initially diagnosed of PNTs, doctors need to make a quick and accurate judgement of the disease condition through the basic clinical characteristics, imaging and bloodwork, and then determine the suitable diagnosis and treatment strategies. The published reports confirmed the numerous clinical and biological differences between diverse histotypes of PNTs. However, in clinical practice, there were no clear indicators and processes to differentiate malignant PNTs from GNs before histological examination. In addition, we often experience the uncertain pathological findings of biopsies for differential diagnostic between malignant PNTs and GNs especially when core needle biopsies were performed because biopsies could not reflect all histological features of the huge tumors. In previous studies it has been demonstrated that sometimes it has a malignant PNT (GNBi or GNBn) on surgery following initial biopsy of GN^9^. Therefore, it is necessary to build discriminative models based on the clinical and biological factors of PNTs to guide the clinical differential diagnosis and treatment.

In this study, we compare the characteristics between malignant PNTs and GNs. Meanwhile, we further find the best factors to discriminate malignant PNTs in all patients with PNTs, in order to recognize malignant PNTs as soon as possible and conduct a complete evaluation of tumor and give the personalized systemic therapy for these patients.

## Patients and Methods

### Patient information

A total of 476 patients diagnosed with PNTs, who were histopathologically proven in Beijing Children’s Hospital (BCH) Affiliated to the Capital Medical University between January 2013 and January 2018 were included retrospectively in this study. The basic information of patients were collected by referring to medical records, including age, sex, laboratory, imaging, and histopathological results. The initial diagnosis of malignant PNT was established by histology, as well as typical bone marrow metastasis in combination with abnormal catecholamines according to the International Neuroblastoma Staging System (INSS) criteria^10^. In special cases of seriously ill patients without bone marrow metastasis, the initial clinical diagnosis was established by typical tumor localisation with typical metastases (such as bone, liver, lymph node, skin and etc) detected by fluorine-18-fluoro-2-deoxy-D-glucose positron emission tomography/computed tomography (^18^F-FDG PET/CT), and combined with abnormal tumor marker levels. However, the histology results of delayed surgery confirmed that these patients were malignant PNTs. All GNs were diagnosed by histology. All methods were carried out in accordance with relevant guidelines and regulations and the study has been approval by the Medical Ethics Committee of Beijing Children’s Hospital (2017-k-89). A waiver of consent was awarded to conduct analyses on the study.

### Laboratory analysis

Laboratory analysis was done prior to treatment and the interval between laboratory tests and the biopsy was less than 15 days. Urinary vanillylmandelic acid (VMA) of 24 h was analyzed by High Performance Liquid Chromatography (HPLC) technique. Lactate dehydrogenase (LDH), neuron-specific enolase (NSE), and ferritin were determined on serum, using routine clinical chemistry laboratory methods.

### Imaging analysis

The radiology reports of ultrasound, computed tomography (CT), magnetic resonance imaging (MRI), and/or ^18^F-FDG PET/CT were collected and analyzed retrospectively. Tumor lesions were identified on images at the primary site as any mass adjacent to the adrenal glands, in a paravertebral location, or in other tumor-typical regions of the head, thorax, abdomen or pelvis. According to the Response Evaluation Criteria in Solid Tumors (RECIST) guidance, published in 2000 and revised in 2009, tumor evaluation relied on measurement of nonnodal target lesions based on the longest single diameter^11^. Maximum diameters of primary tumors in CT and/or MRI were analyzed for all patients. Furthermore, by reference to the Image-Defined Risk Factors (IDRFs) in neuroblastic tumors^12^, we analyzed the relationship between primary tumor and important blood vessels (such as carotid, vertebral artery, subclavian vessels and internal jugular vein in neck; aorta, vena cava and/or major branches in thorax; aorta, vena cava, coeliac axis, superior mesenteric artery, and/or major branches, renal vessels in abdomen; iliac vessels in pelvis), which was one of the most important factors in determining whether the upfront tumor resection could be performed, and the relationship was classified as encasement, displacement, none, and unknown.

### Histopathology

Tumors were classified in accordance with the International Neuroblastoma Pathology Classification System (INPC)^13^. Histologically, the balance between neural-type cells and Schwann-type (Schwannian) cells helped to categorize the tumor as NB, GNB, or GN, and morphologic indicators [grade of neuroblastic differentiation: undifferentiated, poorly differentiated and differentiating; and mitosis-karyorrhexis index (MKI): low, intermediate, and high MKI] for tumors were tested.

### Statistical analysis

Statistical analysis was performed using SAS 9.4. Four hundred and seventy-six patients were included in this study, including 345 patients (January 2013 to January 2017) for model development and 131 patients (January 2017 to January 2018) for external validation. Clinical and biological factors of PNTs were analyzed in 345 patients with PNTs (January 2013 to January 2017). Tumor markers with abnormal distribution were presented as median and interquartile range. Mann-Whiney U test of tumor markers was used for comparing between malignant PNT subgroup and GN subgroup. Receiver operating characteristic (ROC) curve analysis was performed to determine the most appropriate cut-off values for age, tumor marker and maximum diameter of primary tumors. Univariate and multivariate logistic regression analyses were conducted to select potentially useful characteristics for discrimination of histopathology subtypes. The area under the receiver-operating-characteristic (AUC-ROC) curves of the model were calculated. External validation was conducted to verify the model effect. Back substitution test was conducted in patients with inconsistent pathological results of biopsy and surgery. *P* < 0.05 was considered to be statistically significant.

### Data availability

The datasets used and analyzed during the current study are available from the corresponding author on reasonable request.

## Results

### Patients and tumor characteristics

The main characteristics of 345 patients diagnosed between January 2013 and January 2017 with PNTs were analyzed, including 260 malignant PNTs (75.4%) and 85 GNs (24.6%). Among the 260 malignant PNTs, 52 cases were initially diagnosed by upfront surgery, 28 cases by biopsy of surgery, 28 cases by needle core biopsy, 117 cases by typical bone marrow metastasis in combination with abnormal catecholamines and 35 cases were diagnosed by delayed surgery after the initial clinical diagnosis. All 85 GNs were diagnosed by histology of biopsy after surgery. Compared with the main characteristics of GN, those of malignant PNTs showed significant differences (Table 1). Patients with malignant PNTs were more frequently male (54.2% vs 40%) and were diagnosed at a younger median age (33 vs 72 months); the tumor was more often in the adrenal (56.5% vs 17.6%) and less frequently in the retroperitoneal (11.9% vs 28.2%). Moreover, the tumor markers of malignant PNTs were significantly differed from GNs, since they almost had elevated values of serum NSE (*P* < 0.0001), ferritin (*P* = 0.0024), LDH (*P* < 0.0001), and urinary VMA (*P* = 0.0009). By comparing the imaging findings, we found that, maximum diameter of primary tumor in malignant PNTs was larger than in GNs (*P* = 0.0002), and malignant PNTs were more prone to encase important blood vessels (72.3% vs 24.7%, *P* < 0.0001). Furthermore, statistically significant variables in Table 1 were included into multivariate analysis, and continuous variables were determined the most appropriate cut-off values by ROC curve analysis.

**Table 1:** Characteristics of patients with malignant PNTs and GN.

Variables		Malignant PNTs^a,b^	GN	Results	*P*
n		260	85		
Gender	Male	141 (54.2%)	34 (40.0%)	5.190	0.022
	Female	119 (45.8%)	51 (60.0%)		
Primary site	Adrenal	147 (56.5%)	15 (17.6%)	43.941	<0.001
	Retroperitoneal	31 (11.9%)	24 (28.2%)		
	Thoracic	76 (29.2%)	41 (48.2%)		
	Pelvic	5 (1.9%)	5 (5.9%)		
The relationship between primary tumor and blood vessels	Encasement	188 (72.3%)	21 (24.7%)	63.916	<0.001
	Displacement	34 (13.1%)	31 (36.5%)		
	None	34 (13.1%)	33 (38.8%)		
	Unknown	4 (1.5%)	0 (0.0%)		
Age (months)		33.00 (18.00, 50.00)	72.00 (53.50, 97.50)	9.985	<0.001
Maximum diameter of primary tumor (cm)		8.50 (6.00, 12.23)	6.71(4.80, 8.80)	−3.779	<0.001
NSE (ng/mL)^c^		269.00 (63.10, 370.00)	21.25 (18.70, 24.90)	−11.473	<0.001
Ferritin (ng/mL)		208.70 (60.10, 453.40)	44.80 (21.98, 81.93)	−3.030	0.002
LDH (U/L)		572.50 (333.00, 1203.75)	222.00 (199.00, 255.00)	−11.470	<0.001
VMA (mg/24 h)		13.92 (5.45, 37.53)	4.99 (3.60, 7.58)	−3.320	<0.001

^a^Continuous variables were presented as median and interquartile range;

^b^Classification variables were presented as numbers (percent);

^c^Reference ranges of tumor markers: serum NSE ≤ 25 ng/mL; serum ferritin 6 ng/mL – 159 ng/mL; serum LDH 110 U/L – 295 U/L; urinary VMA ≤ 13.6 mg/24 h

PNTs: peripheral neuroblastic tumors; GN: ganglioneuroma; NSE: neuron-specific enolase; VMA: vanillylmandelic acid; LDH: lactate dehydrogenase.

### Determination of cut off value

**Figure 1:**
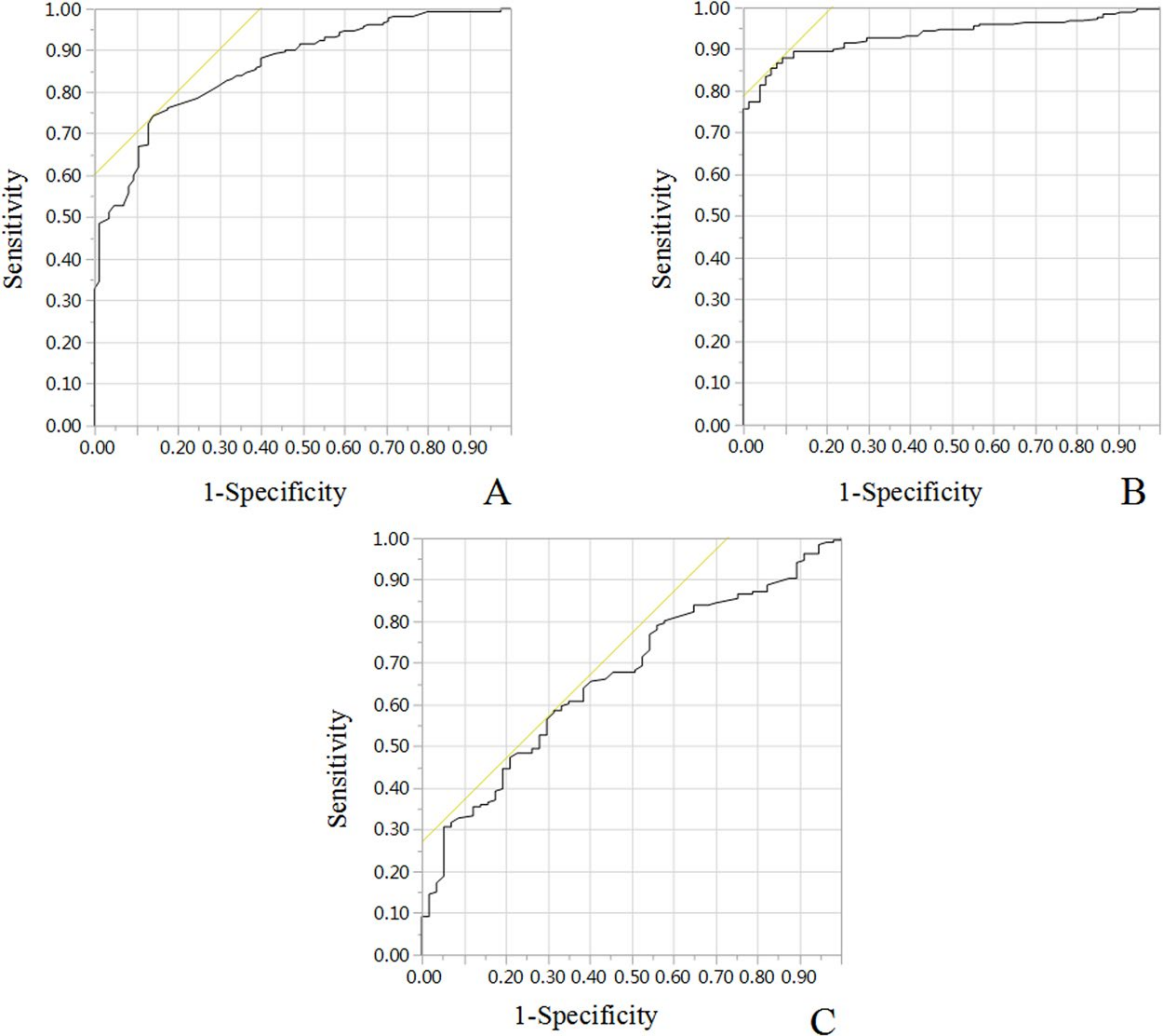
ROC curve analyses. Stratification value of (**A**) age; (**B**) serum NSE; (**C**) maximum diameter of primary tumor were calculated by ROC curve analyses.

According to the maximum joint sensitivity and specificity values, stratification value of age, NSE, maximum diameter of primary tumor were calculated by ROC curve analyses. The cut off value for the characteristics above were 49 (months), 33 (ng/mL), and 7.97 (cm), respectively (Table 2 and Fig. 1).

**Table 2:** Results of the ROC analysis.

Variables	AUC-ROC	Cut-off value
Age	0.86	49.00 (months)
Serum NSE	0.93	33.00 (ng/mL)
Maximum diameter of primary tumor	0.67	7.97 (cm)

AUC-ROC: area under the receiver-operating-characteristic; NSE: neuron-specific enolase.

### Multivariate logistic regression analysis

**Figure 2:**
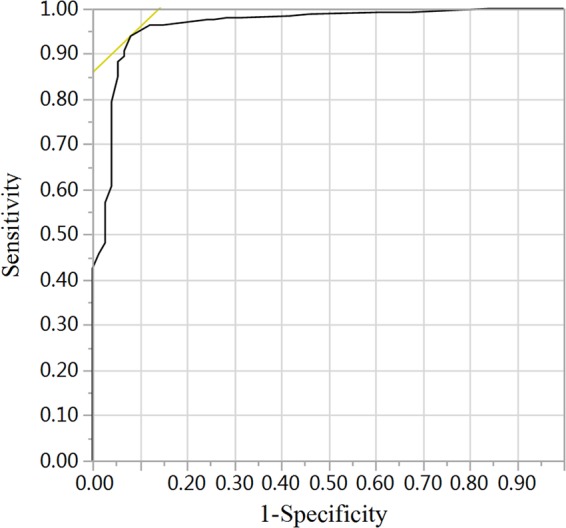
ROC for discrimination of malignant PNTs. Combined age ≤ 49 months, primary site of adrenal and thoracic, level of serum NSE > 33 ng/mL, and tumor encasing blood vessels to discriminate of malignant PNTs and the AUC of the discrimination model was 0.96.

To further identify the characteristics linked to histopathology, multivariate analysis was conducted. After multivariate analysis, we found that age, primary site, serum NSE and the relationship between primary tumor and blood vessels were significant independent factors to discriminate histopathology between malignant PNTs and GNs. The adjusted OR (odds ratio) of the characteristics above were shown in Table 3. The results showed that there were four vital discrimination factors of malignant PNTs as followed, age ≤ 49 months, primary site of adrenal and thoracic, level of serum NSE > 33 ng/mL, and tumor encasing blood vessels, especially level of serum NSE > 33 ng/mL [OR, 36.154 (10.681, 122.379)] and age ≤ 49 months [OR, 19.049 (6.470, 56.088)]. The AUC of the model was 0.96 (Fig. 2). The accuracy rate, sensitivity and specificity were 93.4%, 96.3% and 83.8%, respectively. In our algorithm, small proportion (21 out of 317, 6.6%) of patients were misclassified, including 9 malignant PNTs missclassified as GNs and 12 GNs missclassified as malignant PNTs (Table 4).

**Table 3:** Multivariate logistic regression analysis of prediction of malignant PNTs.

Characteristics	Estimate	Standard Error	Wald	*P*	OR
Intercept	−10.73				
Age > 49 (months)	Ref				
Age ≤ 49 (months)	2.95	0.55	28.61	<0.001	19.049 (6.470, 56.088)
Primary site of retroperitoneal	Ref				
Primary site of adrenal	2.47	0.85	8.48	0.004	11.806 (2.242, 62.179)
Primary site of thoracic	1.99	0.74	7.25	0.007	7.280 (1.717, 30.869)
Primary site of pelvic	1.55	1.35	1.31	0.253	4.695 (0.331, 66.507)
Serum NSE ≤ 33 (ng/mL)	Ref				
Serum NSE > 33 (ng/mL)	3.59	0.62	33.26	<0.001	36.154 (10.681, 122.379)
None relationship between primary tumor and blood vessels	Ref				
Tumor encasing blood vessels	1.82	0.60	9.49	0.002	6.163 (1.937, 19.608)
Tumor displacing blood vessels	−0.21	0.65	0.10	0.751	0.813 (0.227, 2.916)

PNTs: peripheral neuroblastic tumors; OR, odds ratio; NSE, neuron-specific enolase.

**Table 4:** Comparison between results using the model produced and the actual diagnosis produced by pathology.

Practice^a^	Discrimination^b^	
	Malignant PNTs	GN
Malignant PNTs	234	9
GN	12	62

^a^Practice represents the truth observed by pathology.

^b^Discrimination represents the predicted value from the model.

PNTs: peripheral neuroblastic tumors; GN: ganglioneuroma.

### External validation

One hundred and thirty-one patients with PNTs diagnosed between January 2017 and January 2018 were collected to conduct the external validation of the model. The accuracy rate was 97.0% (127/131). Four patients were misdiscriminated, including 2 malignant PNTs and 2 GNs.

### Patients with inconsistent pathological results of biopsy and surgery

Four hundred and seventy-six patients were included in this study, containing 345 patients for model development and 131 patients for external validation. Among the 476 patients of this study, there were 12 patients had inconsistent pathological results of biopsy and surgery. The biopsy results of these patients showed benign tumor of GN, but the final surgery pathological results were malignant PNTs (Table 5). Thus, in order to verify the model effects of these patients, back substitution was conducted to discriminate malignant PNTs. The predicted results of 11 patients were consistent with the practice pathological results (all predicted values were > 0.90), which proved that the discrimination model was of high accuracy, even applied to cases that were difficult to identify of pathological examination.

**Table 5:** Discrimination results of model in patients with inconsistent pathological results of biopsy and surgery.

Patient	Age (months)	Serum NSE (ng/mL)	Primary site	Relationship between primary tumor and blood vessels	Biopsy method	Surgical pathology result	Predicted
1	37	159.0	Adrenal	Encasing	Surgery (skin metastasis)	NB	0.99868
2	45	21.4	Thoracic	Encasing	Core needle^a^	GNBi	0.92828
3	40	70.4	Adrenal	Encasing	Core needle	GNBi	0.99868
4	15	43.2	Adrenal	None	Surgery (cervical lymph node metastasis)	NB	0.99194
5	41	146.4	Adrenal	None	Core needle	NB	0.99194
6	75	28.0	Adrenal	None	Core needle	GNBi	0.15166
7	11	98.0	Thoracic	Encasing	Core needle	GNBn	0.99787
8	18	63.2	Retroperitoneal	Encasing	Core needle	NB	0.98468
9	78	320.1	Adrenal	Encasing	Core needle	NB	0.97551
10	39	52.5	Retroperitoneal	Encasing	Core needle	GNBi	0.98468
11	28	370.0	Adrenal	Encasing	Core needle	NB	0.99868
12	77	63.3	Adrenal	Encasing	Core needle	GNBn	0.97551

^a^Core needle represented the core needle biopsy to primary tumor.

PNTs: peripheral neuroblastic tumors; GN: ganglioneuroma; NSE: neuron-specific enolase; NB: neuroblastoma; GNBn: ganglioneuroblastoma nodular; GNBi: ganglioneuroblastoma intermixed.

## Discussion

Malignant PNTs and GNs are differed in numerous clinical and biological characteristics, and thus the treatment strategies are not exactly the same. The modern comprehensive treatment of malignant PNTs depends on risk group which is determined on patient age, tumor stage, histopathological appearance, and molecular biological factors. On the contrary, GN is considered to be a benign tumor of neural crest origin^13^ and tumor resection or observation is recommended^3,14^. However, in clinical practice, there were no clear indicators and processes to differentiate malignant PNTs from GNs before histological examination. In addition, sometimes we experience the uncertain findings of biopsies for diagnosis of different histopathology subgroups especially when core needle biopsies were performed. Therefore, it is necessary to build discriminative models to guide the clinical diagnosis and treatment. Thus, the result of this study may help us to discriminate PNTs by combining clinical and biological characteristics, and then give different evaluation and treatment strategies to malignant PNTs and GNs respectively.

Former studies found that compared to GNs, malignant PNTs were characterized by a significant increase in tumor marker levels [Serum NSE, ferritin, LDH, urinary VMA and homovanillic acid (HVA)], disseminated disease, younger age, more common primary site of abdomen and abnormal cytogenetics and molecular genetics^8,9^. As a relative specific tumor marker of PNTs, a large number of studies have analyzed the association between NSE level and initial diagnosis^10^, response assessment, INPC group^15^, tumor relapse or progression^16^, and prognosis^17,18^, and we found the importance of NSE level in the discriminative of different histopathology subgroups of PNTs. In terms of the primary tumor site, Vo *et al*.^19^ found that malignant PNTs were more often in adrenal, but Decarolis *et al*.^8^ found that GNs were less frequently in adrenal. In addition, in CT and MRI images, malignant PNTs mostly located in adrenal or in a paravertebral location with vascular embedding, local tissues metastasis and abundant blood supplying, which were reflections of infiltration and invasion features of malignant tumors. Whereas GN had relatively regular morphology and clear boundary, with vascular displacement as a primary manifestation^20,21^. As for the prognosis, malignant PNTs and GNs were strikingly distinct. Even after aggressive treatment, the prognosis of malignant PNTs especially late stage and high risk patients was still unsatisfactory. On the country, GN could get a good therapeutic effect only by surgical resection without any cytotoxic treatment^3,14^, and few patients had tumor recurrence or residual lesions of malignant transformation after incomplete resection^9,22^.

Some researches hypothesized that a good part of GN originated from NB. This theory has first been suggested by Cushing in 1926^23^ and has since been suspected repeatedly for stage 4S^24–26^ as well as for localized NB^27^. An indirect support for this hypothesis can be seen by our observation that patients with GNs were older at diagnosis than those with malignant PNTs (median age of 72 vs 33 months). And former reports showed that the grade of neuroblastic differentiation increased with the median age at diagnosis and this was in line with the theory that mature PNTs might just had enough time to mature *in situ* before they were discovered^8^. However, whether this is true for all GNs, as proposed by Shimada^1^, still remains unproven.

NB, GNB, and GN represent tumors of neural crest origin with a continuous spectrum of neuronal maturation. In fact, maturation within a single tumor may not be homogeneous. This may complicate histopathological analysis, and biopsies especially needle core biopsies may not be representative. Although core needle biopsies are usually adequate to identify a tumor as neuroblastic in origin, they do not provide sufficient information about higher-order architecture and regional histological variation that could affect INPC classification and assignment of risk grouping and treatment protocol^28^. In this study, we can find that the biopsy of 12 patients suggested GN, whereas histological examination after total resection showed GNB or NB. We further used the model to discriminate malignant PNTs of these patients and found that only one patient’s predicted result was incorrect. The core needle biopsy showed GN whereas the eventually surgical histological examination revealed a localized GNBi tumor.

To sum up, the results allow the conclusion that combined age ≤ 49 months, primary site of adrenal and thoracic, level of serum NSE > 33 ng/mL, and tumor encasing blood vessels can help us to discriminate malignant PNTs in all PNTs with high accuracy. The result of external validation of the model showed a highly accuracy for the identification of cases, not only in external validity, but also in patients with inconsistent pathological results of biopsy and surgery. However, the generalizability of the model in other ethnicity is unknown. This result may lay the foundation for the development of differential diagnosis and identification strategies to malignant PNTs and GN in the future, and it is helpful to offer different evaluation and treatment strategies to those patients respectively.
